# Determining the Shear Strength and Permeability of Soils for Engineering of New Paddy Field Construction in a Hilly Mountainous Region of Southwestern China

**DOI:** 10.3390/ijerph17051555

**Published:** 2020-02-28

**Authors:** Zhen Han, Jiangwen Li, Pengfei Gao, Bangwei Huang, Jiupai Ni, Chaofu Wei

**Affiliations:** 1 College of Resources and Environment, Southwest University, Chongqing 400715, China; m13658372800@163.com (Z.H.); ljw0638@163.com (J.L.); 18404983640@163.com (P.G.); hbw2020212@163.com (B.H.); nijiupai@163.com (J.N.); 2 Key Laboratory of Arable Land Conservation (Southwestern China), Ministry of Agriculture, Chongqing 400715, China; 3 State Cultivation Base of Eco-agriculture for Southwest Mountainous Land, Southwest University, Chongqing 400715, China

**Keywords:** new paddy field construction, shear strength, cohesion, water content, permeability property

## Abstract

As a constructed wetland ecosystem, paddy field plays an irreplaceable role in flood storage and detention, groundwater replenishment, environmental protection, and ecological balance maintenance. New paddy field construction can give full play to the production and ecological functions of paddy field and can adjust the development structure of the agricultural industry effectively. The soil properties of shear strength and permeability, which provide a theoretical basis for engineering design, construction, and post-operation, are important indexes in the site selection of new paddy field. The shear strength and permeability properties of soils from different land use types (vegetable field, gentle slope dryland, corn field, grapery, and abandoned dryland) for engineering new paddy field construction were investigated in this study. The results showed that the soil water content had a significant effect on the soil shear strength, internal friction angle, and cohesion. The total pressure required for soil destruction decreased with increasing water content under the same vertical pressure, resulting in easier destruction of soils. The internal friction angle decreased with increasing soil water content, and the soil cohesion first increased and then decreased with increasing soil water content. Considering that paddy fields were flooded for a long time, the soil strength properties had certain water sensitivity. Effective measures must be taken to reduce the change in soil water content, so as to ensure the stability of the embankment foundation, roadside ditch foundation, and cutting slope. In addition, the influence of changing soil water content on the strength properties of paddy soils should be fully considered in engineering design and construction, and the soil bulk density at the plough pan should reach at least 1.5 g cm^−3^ or more to ensure better water retention and the anti-seepage function of paddy field. The study can provide construction technology for engineering new paddy field construction in a hilly mountainous region of southwestern China.

## 1. Introduction

Rice, mostly as paddy, grows on approximately 157 × 10^6^ million ha of the world’s surface, which makes up the world’s most important anthrosol for food production and security [1,2,3,4,5,6]. It is estimated that a 40% increase in rice production is needed to meet the surging demand from the rapidly increasing population by the end of 2030 [7,8]. China is the world’s largest rice producer, accounting for 30% of total world production, with 29, 850 ha [9], followed by India (22%), Indonesia (9%), and Bangladesh (7%) [3,10]. On the other hand, paddy field, as constructed wetland ecosystems, plays an irreplaceable role in flood storage and detention, groundwater replenishment, environmental protection, and ecological balance maintenance [3]. Therefore, paddy field is environmentally friendly and belongs to sustainable land use type [11].

**Figure 1:**
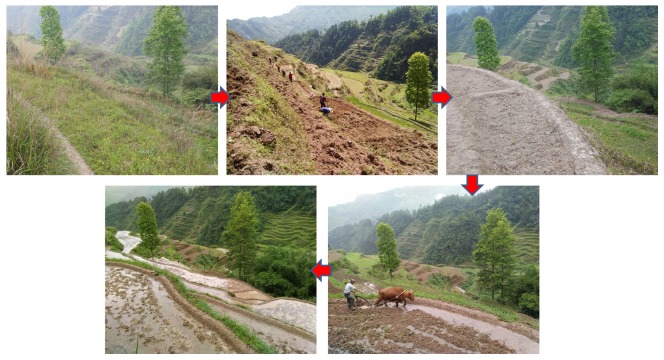
The engineering processes of new paddy field construction.

Paddy field originated 7000–6000 years ago, and the evolution of paddy field from small to large and irregular to standard had lasted for more than 2000 years [11]. New paddy field construction was a concept put forward after the 1990s to increase paddy field planting area and grain yield by changing dryland to paddy field (Figure 1). Paddy field is designed reasonably according to the natural and topographical conditions of the planning area, the local villagers’ farming habits and the structure of the agricultural planting varieties. New paddy field construction is an important part of land consolidation and an effective measure to improve the quality of cultivated lands. Over the years, many places adopted a way of emphasizing development to occupy a large amount of fertile lands. It required that in the process of land consolidation, the lands should be converted into high-quality farmland, especially paddy field, so as to meet the demand of balance of occupation and compensation. Therefore, the engineering of new paddy field construction had huge market demand and economic benefits. However, most of the previous engineering constructions only rely on accumulated experience and lack some scientific basis in determining the engineering parameters. To avoid waste of construction, it is necessary to focus on the physical properties of soils for new paddy field construction.

In view of the relative lack of technical research on new paddy field construction and the rare examples of practical application, it is urgent to integrate and innovate the research and breakthroughs on key technologies of new paddy field construction. Among them, it is very important and necessary to study the stability of new paddy field construction. Soil shear strength is a useful dynamic measure for evaluating the soil stability of engineering technology [12,13,14] and an important input parameter in the design of precise tillage and large agricultural instruments. The basic reason for the instability of the paddy field surface is that the shear stress on a certain surface inside the soil reaches its shear strength, thus destroying the stable balance. There are two reasons why the shear stress reaches the shear strength: first, the increasing shear stress, such as the increase in the soil water content increases the soil body weight, and rainfall produces seepage forces and hydrodynamic pressure. Second, the shear strength of the soil decreases, such as the increase in the pore water stress, weather cracking, freeze-thaw, and creep of cohesive soil caused by climate change [15]. In addition, the bearing capacity of the foundation, the earth pressure of the retaining wall, and the stability of the soil slope are directly related to the shear strength of the soils. The cohesion and internal friction angle are commonly used in engineering design to measure changes in the soil shear strength. In particular, the alteration of hydrologic conditions is the most important factor in bonding force variation, thus affecting the shear strength [13,16]. The effect of the water content on shear strength, as one of the hot topics in soil mechanics and land consolidation engineering, has been widely investigated in recent years [13,17]. However, different results regarding the relationship between the shear strength properties and the water content have been reported according to locations, soils, and experimental designs [18,19]. The widely accepted conclusion is that the shear strength reached its peak at a particular water content [20]. In addition, soil permeability has always been an important fertility index of high-yield soils, and the infiltration characteristics of the plough pan are also the key factors that determine the stability of paddy field [21].

Paddy field is high-yield cultivated land and also an efficient constructed wetland system with ecological functions that cannot be ignored. However, the Three Gorges Reservoir Area, as an independent ecological conservation area, has seen an increasingly prominent phenomenon of farmland changing from water to drought, resulting in a large reduction in paddy field and serious damage to the ecological environment in some areas. Therefore, the construction of new paddy field on agricultural farmlands can give full play to the production and ecology of paddy field, can effectively adjust the development structure of agricultural industries, and can meet the needs of the development of ecological agriculture. It is of great significance to promote the sustainable development of paddy field and even cultivated land resources. The shear strength and permeability properties of soils are of great significance in foundation design, retaining wall design, slope stability evaluation, and other issues related to soil stability and strength evaluation. Therefore, the analysis of soil-related problems cannot be separated from the shear strength and permeability properties of soils. They are also important indexes in the site selection of new paddy field construction and provide scientific reference for the engineering design, construction, and post-operation of paddy field. The main objectives of this study were (i) to explore the mechanism of change in soil shear strength with vertical pressure and soil water content under different land use types; (ii) to determine the soil bulk density at the plough pan, ensuring better water retention and the anti-seepage function of paddy field; and (iii) to propose engineering technology for new paddy field construction in a hilly mountainous region of southwestern China.

## 2. Materials and Methods

### 2.1. Study Area and Distribution of Sample Points

**Figure 2:**
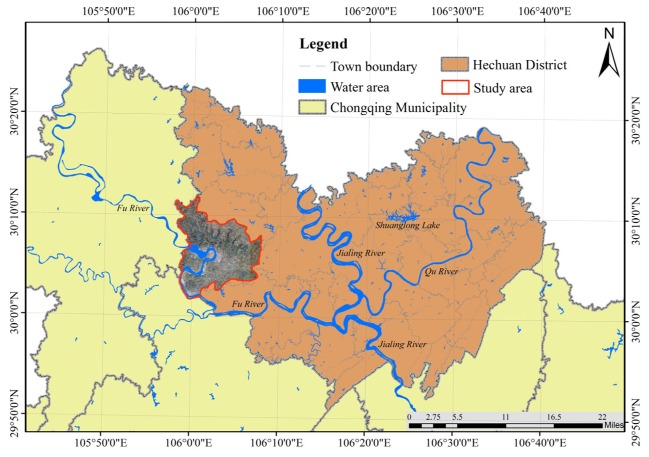
The study area.

The study area is located in Hechuan District (30° 5′ N, 106° 4′ E, and 258 m above mean sea level) in Chongqing, China (Figure 2). It has a subtropical humid climate with a mean annual temperature of 17.8 °C and a mean annual precipitation of 1137 mm that is distributed unevenly throughout the year. Most rainstorms occur between May and October, and the amount of precipitation during this period accounts for ~80% of the total annual precipitation. The test soils, classified as regosols in the FAO Taxonomy or entisols in the USDA Taxonomy, are formed from purple rocks or weathering products and are mainly distributed in the sichuan basin of southwestern China [22,23].

**Figure 3:**
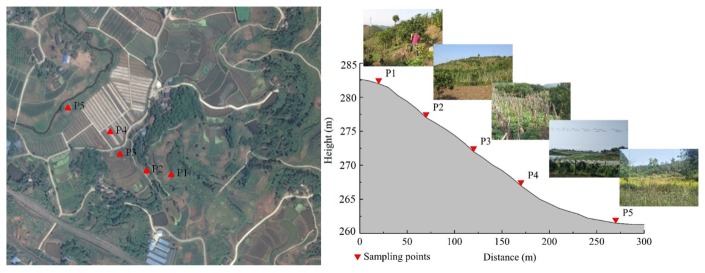
The distribution of sample points (P1-Vegetable field, P2-Gentle slope dryland, P3-Corn field, P4-Grapery, and P5-Abandoned dryland).

The planned scale of new paddy field construction in the study area was 11.89 hm^2^ and mainly included the following five land use types: P1 represents vegetable fields (2.29 hm^2^), P2 represents gentle slope dryland (2.69 hm^2^), P3 represents corn fields (2.48 hm^2^), P4 represents graperies (2.26 hm^2^), and P5 represents abandoned dryland (2.17 hm^2^). Soil samples were collected from the five land use types above, which were distributed in different positions on the slope shown in Figure 3.

### 2.2. Sampling and Physical Properties of the Test Soils

Ten kilograms of soil samples with a depth of 5–20 cm were collected at each sample point in the study area to remove large gravel blocks, animal and plant residues, and other impurities. The samples were air dried in a ventilated environment, then grinding and sieving were carried out to prepare the soil samples with a particle size of 2 mm. Soil particle composition, bulk density, and rock fragment content were determined by the pipette method [24], cutting-ring method, and water washing method [25], respectively. Soil liquid plastic limit was determined by the STYS-1 digital display liquid plastic limit joint tester. The physical properties of the soils at different sampling points were shown in Table 1 and Table A1.

### 2.3. The Determination of Permeability Coefficient and Shear Strength Characteristics

The soil permeability coefficient was measured by using a TST-55 permeameter (variable-head permeameter) produced by Nanjing Soil Instrument Factory, in which the bulk density was set to six gradients (1.2, 1.3, 1.4, 1.5, 1.6, and 1.7 g cm^−3^). To determine the soil shear strength under different water contents, the air-dried soil was configured with seven water content gradients (7%, 10%, 13%, 16%, 19%, 22%, and 25%). The dry density of the test soil was set as 1.50 g cm^−3^. The shear strength parameters were measured by a ZJ strain-controlled direct shear apparatus (quadruple shear) manufactured by a Nanjing Soil Instrument Plant. According to the preset water content and dry density, the soil was formed into a specimen with a diameter of 61.8 mm and a height of 20 mm by using a compactor. The soil samples with the same water content were repeated three times, and the error of the dry density was 0.1 g cm^−3^. The vertical pressures of 100, 200, 300, and 400 kPa were applied. The shear rate was controlled to 0.8 mm min^−1^ by means of fast shear. The test data were collected by the microcomputer control data processing system of the Zhilong geotechnical test provided by the ZJ strain control direct shear instrument. Failure was taken as the peak shear stress attained, or in the absence of a peak condition, the shear test was stopped at 10% of the relative lateral displacement of the specimen.

## 3. Results

### 3.1. The Relationship between the Shear Stress and Displacement

**Figure 4:**
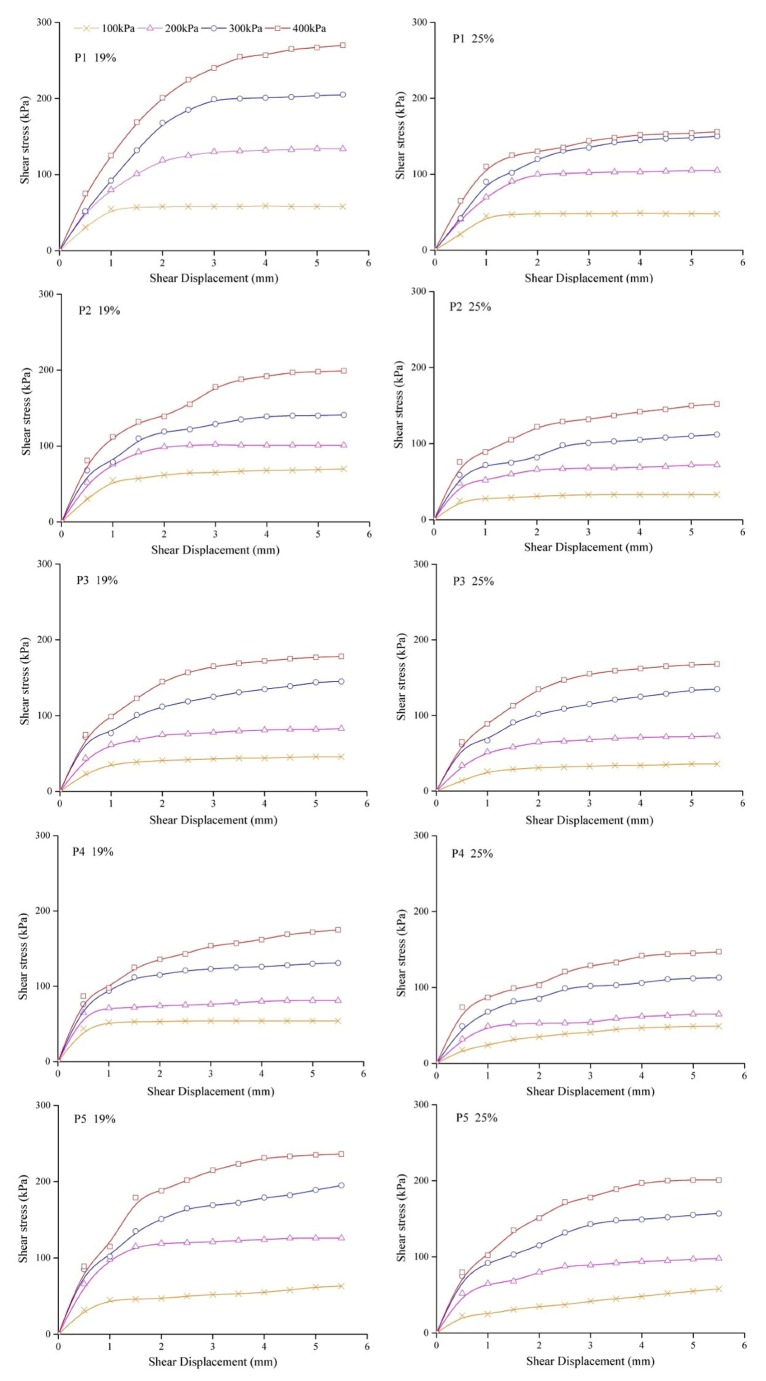
Typical shear-strength-displacement curves for different sample points (P1, P2, P3, P4, and P5) at different moisture contents (19% and 25%) and net normal stresses (100, 200, 300, and 400 kPa).

The shear strength of the soils under seven water contents and four vertical pressures was measured by a strain-controlled direct shear apparatus. The stress–strain relationship curves under the water content of 19% and 25% were selected to analyze the characteristics of the shear process (Figure 4). The shear stress increased rapidly to a certain degree called the residual strength. The shear stress increased with increasing shear displacement, especially at lower water contents. The higher the vertical pressure, the greater the shear stress. When the vertical pressure was 100 or 200 kPa and the shear displacement was less than 1 mm, the shear stress increased rapidly with increasing shear displacement. When the vertical pressure was 300 or 400 kPa and the shear displacement was less than 2 mm, the shear stress also increased with increasing shear displacement. When the shear displacement under small pressure was larger than 1 mm or the shear displacement under large pressure was larger than 2 mm, the rate of change in the shear stress with shear displacement decreased, and the shear stress and shear displacement showed a linear increasing trend. When the shear displacement increased to 4 mm, the increase in the shear stress was very small and tended to be stable, especially when the vertical pressure was relatively small with no obvious peak in the whole shear process. Therefore, the shear stress corresponding to the shear displacement of 4 mm was the shear strength value at this water content and vertical pressure.

### 3.2. Effect of Water Content and Vertical Pressure on Shear Strength

**Figure 5:**
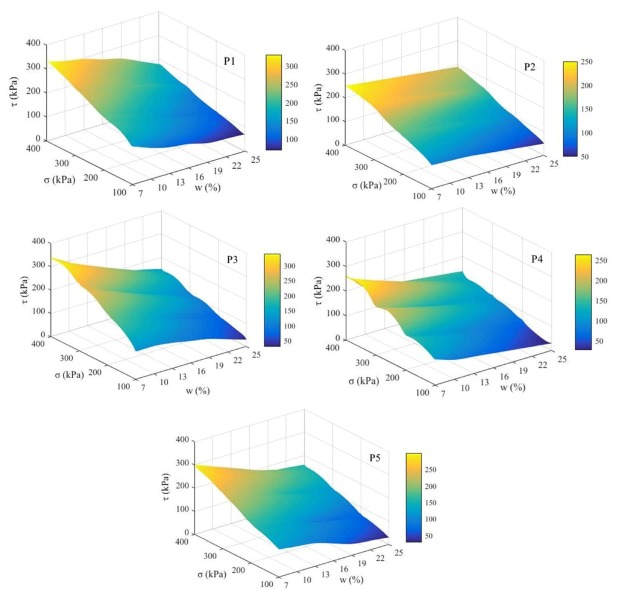
The variation in soil shear strength under different soil water contents and vertical pressures (τ, σ, and w represent the soil shear strength, vertical pressure, and soil water content, respectively).

A direct shear test is used to determine the relationship between the shear stress and shear displacement under different vertical loads. The shear strength was negatively correlated with the soil water content and positively correlated with the vertical pressure in Figure 5. Under the same vertical pressure, with the increase in the water content, the shear strength gradually decreased, and the range of the soil shear strength at the five locations was different in the process of increasing the soil water content from 7% to 25%. When the vertical pressure was 100 kPa, the shear strength was the smallest. When the vertical pressure was 400 kPa, the shear strength was the highest. In addition, when the pressure was 100 and 400 kPa, the ranked shear strengths of the samples were P1 > P3 > P5 > P4 > P2 and P3 > P1 > P5 > P4 > P2, respectively. Moreover, the range of the maximum variation in the soil shear strength of the samples with water content was P3 > P1 > P5 > P4 > P2. In addition, during the change in the vertical pressure from 100 to 400 kPa, the larger the vertical pressure was, the greater the variation in the shear strength with water content.

### 3.3. Water Sensitivity Characteristics of the Shear Strength Parameters

**Figure 6:**
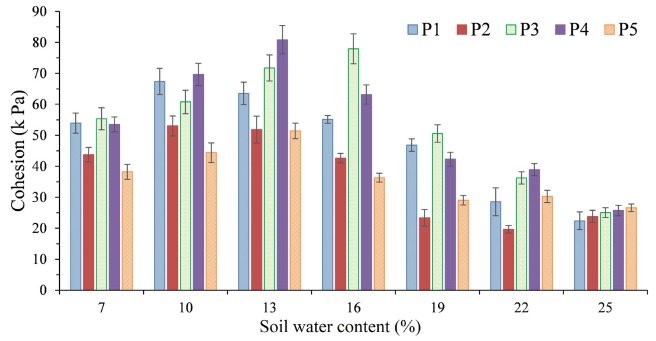
Cohesion with respect to the total stress for different sample points (P1, P2, P3, P4, and P5) at different water contents (7%, 10%, 13%, 16%, 19%, 22%, and 25%).

Cohesion is caused by the gravitational interaction between soil particles, and the water content is the main influencing factor for a particular soil mass. The cohesion of sample P1 first increased and then decreased with increasing water content and had an obvious peak value (Figure 6). When the water content was 10%, the maximum cohesion, 67.39 kPa, was achieved. The cohesion of sample P2 increased first, then decreased and increased again with the increase in water content, with the interval peak value. When the water content was 10%, its cohesion reached a maximum value of 53.02 kPa, and when the water content was 22%, the minimum value was 19.68 kPa. In addition, the cohesion of samples P3 and P4 with the change in water content was basically the same as that of sample P1, showing a trend of first increasing and then decreasing, with an obvious peak value. When the water content was 16% and 13%, the maximum soil cohesion of samples P3 and P4 reached 77.93 and 80.86 kPa, respectively. The cohesion of sample P5 increased rapidly first, then decreased rapidly, increased slowly again, and then decreased slowly with increasing water content. It is noteworthy that the change had two peaks. When the water content was 13%, the cohesion increased rapidly to peak 1 (51.44 kPa) and then decreased rapidly; when the water content was 22%, the cohesion increased slowly to peak 2 (30.26 kPa). In summary, with the change in the water content, the maximum soil cohesion of samples P1 to P5 was 67.39, 53.02, 77.93, 80.86, and 51.44 kPa, and the corresponding water content was 10%, 10%, 16%, 13%, and 13%, respectively. The maximum cohesion was reached at the plastic limit of the soil. When the water content was less than 13%, the cohesion of sample P4 was greater, and then that of sample P3 was larger.

**Figure 7:**
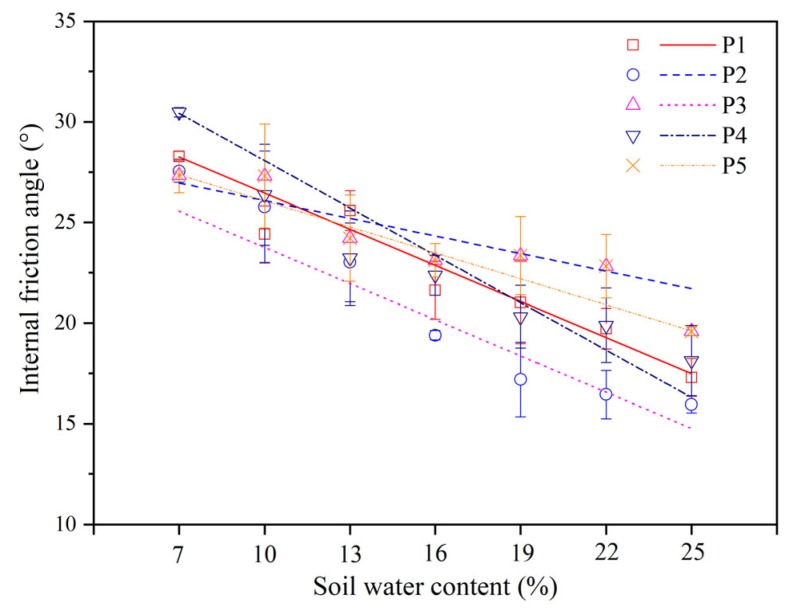
Internal friction angle with respect to the soil water content for different sample points (P1, P2, P3, P4, and P5).

The internal friction angle is mainly caused by the biting interaction among soil particles. Figure 7 showed the relationship between the internal friction angle (*φ*) and the soil water content (*w*). There was a linear negative correlation between the soil water content and internal friction angle in five different locations of the soils. The larger the water content was, the smaller the angle of internal friction. The effect of the water content on the internal friction angle was related to the tillage method. Under different tillage patterns, the effects of the water content on the internal friction angle were P3 > P4 > P1 > P5 > P2. This indicated that the water content had the greatest influence on the internal friction angle of the soils in the corn field. In the whole water content test interval, the maximum values of the soil internal friction angle corresponding to the five sample points (P1, P2, P3, P4, and P5) were 28.28°, 27.01°, 27.55°, 30.48°, and 27.33°, and the minimum values were 17.30°, 21.74°, 15.96°, 18.13°, and 19.59°, respectively.

The internal friction angles of the five sample points had a consistent trend with the change in water content, and the linear negative correlations were shown in Table 2.

The relationship between the soil shear strength and the water content was developed by the Mohr-Coulomb law and is shown in Table 3. The equation is as follows:

(1) τ=σtanφ+c

where *τ*, *σ*, and c represent the soil shear strength (kPa), vertical pressure (kPa), and cohesion (°), respectively.

### 3.4. Permeability Characteristic of the Test Soils

**Figure 8:**
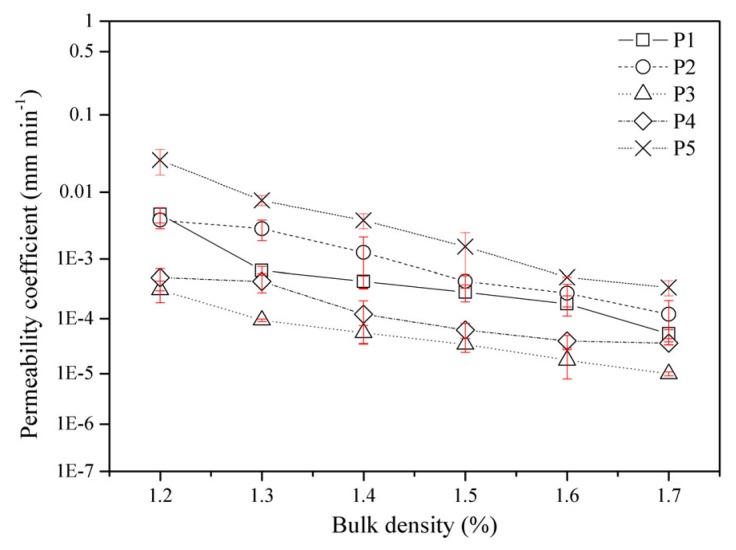
Permeability characteristic for different sample points (P1, P2, P3, P4, and P5) at different bulk densities (1.2, 1.3, 1.4, 1.5, 1.6, and 1.7 g cm^−3^).

The permeability characteristics of the test soils were analyzed under different bulk densities (1.2, 1.3, 1.4, 1.5, 1.6, and 1.7 g cm^−3^) to clarify the key points of plough pan construction as shown in Figure 8. At the same bulk density, the permeability coefficient was related to the land use type. With increasing clay content, the permeability coefficient showed a decreasing trend with the order of P5>P2>P1>P4>P3. Increased bulk density caused increasing soil compactness and decreased porosity. Therefore, the permeability of the soil decreased correspondingly, and the permeability coefficient decreased gradually with a variation range of two to four orders of magnitude. The soil permeability coefficient at different points slowed with decreasing bulk density after the bulk density was 1.5 g cm^−3^, so the soil bulk density at the plough pan should reach at least 1.5 g cm^−3^ or more.

## 4. Discussion

### 4.1. The Shear Strength of the Test Soils Decreases Obviously with Increasing Water Content

The occurrence of soil damage is often caused by the destruction of internal soil particles, and the shear strength is an important indicator reflecting the ability of soil to resist shear damage. The external loads were principally sustained by the large particles through the microscopic force chains formed among the particles. Simultaneously, the evolution of the microstructures was closely linked with the movement of fine particles within the soil skeleton, and the role of the large particles in carrying shear loads relied firmly on the supporting effect of the fine particles [26]. The movement and rearrangement of soil particles are influenced by the water content [27]. In this study, the soil shear strength was significantly influenced by the water content, which is consistent with other studies [13,28,29]. Under the same vertical pressure, the total pressure required for soil destruction decreases with increasing water content, resulting in easier destruction of the soil samples (Figure 4 and Figure 5). The reason for this is that when the soil water content increases, the water forms a lubricant on the surface of the soil particles, resulting in a decrease in the internal friction angle of the soil and a sharp drop in the cohesion between the particles. At the same time, the increase in the water content will thicken the water film around the soil particles, and even free water will appear in the pores of the soil, thus resulting in a decrease in the shear strength of the soil. In addition, soil samples from the purple-soil sloping croplands generally contain many roots and are rich in organic matter. The abundant secretions from root systems promote strong adhesion between the soil particles, leading to enhanced cohesion [30]. Therefore, the shear strengths of samples P1 and P3 were larger than those of the other sample points.

Further on, water content has a significant effect on the internal friction angle and cohesion. In general, the internal friction angle decreased with increasing soil water content in the present study, and the soil cohesion first increased and then decreased with increasing soil water content. It is generally believed that the cohesive force of soil is the result of the comprehensive action of attraction and repulsion between soil particles. In addition to being related to the Coulomb force, Van der Waals force, cementation force, osmotic pressure caused by concentration difference, and soil mineral type, it is also affected by the cohesive force of the water film [31,32]. The water film connection and cementation between soil particles play important roles in the generation of cohesive force, so the cohesive force of soil varies greatly with the water content. When the humidity is low, there is no free water in the soil as thin film of water acts between the soil particles at that time. Its connection and cementation have great influences on the cohesion of the soil, and the contact points between the soil particles are increased such that the attraction between molecules is stronger [29], which prevents the relative movement between soil particles and makes it difficult to separate soil particles bonded together. Considering the interaction force between the soil particles at the microscopic level, the long-range Van der Waals force is the main attraction between the particles. In addition, the effective stress in soil is a mutual attraction [33]. As the water content continuously increases, the attraction gradually increases. This is probably the reason why the cohesion increases with increasing water content when the water content of the tested soil is lower than 13%. When the soil water content increases continuously, the bound water film on the particle surface thickens, the lubrication effect is enhanced, and the frictional resistance of the particle relative to sliding under the action of external force is reduced [34], as is the mutual embedded biting force. The acting force between the soil particles dominated by the Van der Waals force is gradually converted into the electrostatic repulsive force [35]. At the same time, the increase in water content will cause the cementation substances, such as iron oxide, in the tested soil to be dissolved and lose their cohesion force. Therefore, when the water content is greater than the threshold value, the cohesion force decreases with increasing water content [31]. In terms of affecting the internal friction angle, the space between the soil particles increases, and the effective contact area decreases as the water content increases. In addition, the friction between the soil particles changes to friction between the soil particles and the water membrane, meaning the friction coefficient gradually decreases [36], resulting in decreasing internal friction angle. In addition, the internal friction angle is also affected by other factors, including the soil particle components, particle size, and compaction [13].

### 4.2. The Permeability of Paddy Field Soils Has an Important Influence on the Construction and Operational Processes of Engineering Projects

In the actual process of engineering construction, changes in soil water content and seepage state will lead to changes in soil stress, resulting in the collapse or dry shrinkage deformation of soils. For new paddy field construction, field water holding capacity is a basic characteristic of soil moisture retention. To ensure the field water holding characteristics of new paddy fields, anti-seepage engineering is required. The key to preventing infiltration of the field surface lies in the construction of the plough pan. The thickness and compaction degree of the plough pan determine the infiltration coefficient of the field surface. In engineering construction, anti-seepage measures should be taken for fields that do not form a plough layer (anti-seepage layer): first, the topsoil should be stripped to construct a plough layer; second, the stripped surface is cut high and filled with depressions. Comprehensive measures such as beating, settling and tamping (rolling), full coverage tamping, and beating and mud standing are carried out until the anti-seepage requirements are met to reduce the influence of the water content on the stability of the paddy fields. To ensure better water retention and anti-seepage function of the paddy fields, the soil bulk density at the plough pan should reach at least 1.5 g cm^−3^ or more. In addition, soil permeability has always been regarded as an important fertility index of high-yield soil. On one hand, permeability enables the supply of soil oxygen under waterlogged conditions to enable soil microorganisms to carry out normal decomposition activities; on the other hand, toxic and reducing substances generated by decomposition of undesirable gases in the soil are leached out to keep the soil in a healthy environment [21]. However, soil permeability may cause the loss of soil nutrients and affect the stability of engineering works. Therefore, the permeability of the soils of paddy fields has an important influence on the construction and operational processes of engineering projects.

## 5. Conclusions

The shear and permeability properties of soils under different land use types for engineering of new paddy field construction were investigated in a hilly mountainous region in southwestern China. Considering that paddy field is flooded for a long time, the soil strength properties have a certain water sensitivity. The strength of the soil drops sharply when the water content exceeds the limit value. Effective measures must be taken to reduce the change in soil water content so as to ensure the stability of the embankment foundation, roadside ditch foundation, and cutting slope. In addition, the influence of changing soil water content on the strength properties of the paddy soils should be fully considered in engineering design and construction. There is a threshold value for the cohesion with the change in soil water content. When the water content is less than this value, the cohesion increases linearly with increasing water content. When higher than the threshold value, the cohesion decreases linearly and sharply. Therefore, the soil characteristics under the threshold water content should be determined when analyzing the stability of paddy fields. Judging from the influence of the water content on the shear strength and soil cohesion, cohesion is the main reason for the formation of soil strength properties. Since the strength properties of the soils at all the sample points will be reduced after the water content is greater than the limit value, flooding in new paddy field construction should be restricted to reduce the natural water content and allow drying in the field before construction of supporting road projects passing through the paddy field. At the same time, ditches should be cut on both sides of the subgrade to remove surface groundwater as much as possible so that the water content in the paddy field can reach the optimal value, thus ensuring the stability of the subgrade. In addition, the influence of the changing soil water content on the strength properties of paddy soils should be fully considered in engineering design and construction, and the soil bulk density at the plough pan should reach at least 1.5 g cm^−3^ or more to ensure better water retention and anti-seepage function of paddy fields.

The following aspects can be further researched in the future: (i) There are many types of soil, and comparative analysis of soil in different types of engineering areas will play an important role in the engineering of new paddy field construction in southwest mountainous region. (ii) In addition to the factors mentioned in this article, the effects of organic matter, soil microorganisms, and soil temperature conditions on soil stability can also be considered.

## Figures and Tables

**Table 1:** The physical properties of the test soils at different sample points.

Sampling Points	Field Types	Soil Texture	Field Area (hm^2^) /Proportion (%)	Bulk Density (g cm^−3^)*	Rock Fragment Content (%)*	Particles Distribution (%)			Liquid Limit (%)*	Plastic Limit (%)*	Plasticity Index (%)*
						>0.02 mm*	0.02–0.002 mm*	<0.002 mm*			
P1	Vegetable field	Loam	2.29/19.26%	1.34 ± 0.03	5.63 ± 0.08	41.91 ± 2.53	47.93 ± 2.45	10.17 ± 0.08	42.74 ± 0.03	30.71 ± 2.63	12.03 ± 0.11
P2	Gentle slope dryland		2.69/22.62%	1.23 ± 0.02	7.56 ± 0.98	40.89 ± 0.95	49.51 ± 1.15	9.60 ± 0.20	41.56 ± 0.21	31.28 ± 0.89	10.28 ± 0.02
P3	Corn field		2.48/20.86%	1.56 ± 0.05	2.14 ± 0.11	42.83 ± 0.01	34.49 ± 0.65	22.68 ± 0.64	46.28 ± 2.12	29.31 ± 1.45	16.97 ± 0.33
P4	Grapery		2.26/19.01%	1.48 ± 0.03	6.05 ± 0.32	45.78 ± 0.24	33.45 ± 0.85	20.77 ± 2.11	44.23 ± 2.31	30.02 ± 1.33	14.21 ± 0.22
P5	Abandoned land		2.17/18.25%	1.36 ± 0.02	13.39 ± 1.12	41.04 ± 4.81	49.54 ± 4.51	9.42 ± 0.30	40.78 ± 5.23	31.52 ± 1.25	12.26 ± 0.18

* The presented data represent the mean ± the standard deviation and are based on three replicates.

**Table 2:** The relationship between the internal friction angle and water content (7%, 10%, 13%, 16%, 19%, 22%, and 25%) for different sample points (P1, P2, P3, P4 and P5).

Sampling Points	Fitting Equation	Adj-*R^2^*	*F* value	*Prob>F*
P1	*Φ* = −0.60*w* + 32.44	0.98	255.60	1.74 × 10^−5^
P2	*Φ* = −0.29*w* + 29.00	0.99	478.40	3.70 × 10^−6^
P3	*Φ* = −0.60*w* + 29.76	0.87	40.87	1.39 × 10^−3^
P4	*Φ* =−0.78*w* + 35.91	0.97	203.37	3.05 × 10^−5^
P5	*Φ* = −0.43*w* + 30.37	0.97	217.42	2.59 × 10^−5^

**Table 3:** The relationship between the soil shear strength and water content (7%, 10%, 13%, 16%, 19%, 22%, and 25%) for different sample points (P1, P2, P3, P4, and P5).

Sampling Points	Fitting Equation	
	7%≤*w*≤13%	13%≤*w*≤25%
P1	τ=σtan(−0.60w+32.44)+4.80w+52.02	τ=σtan(−0.60w+32.44)−11.65w+ 67.36
P2	τ=σtan(−0.29w+29.00)+4.05w+41.43	τ=σtan(−0.29w +29.00)−6.01w+ 42.40
P3	τ=σtan(−0.60w+29.76)+8.20w+46.24	τ=σtan(−0.60w+29.76)−17.30w+90.70
P4	τ=σtan(−0.78w+35.91)+13.67w+40.70	τ=σtan(−0.78w+35.91)−11.56w+71.41
P5	τ=σtan(−0.43w +30.37)+6.62w+31.45	τ=σtan(−0.43w +30.37)−2.80w+37.53

## Appendix A

**Table A1:** The importation of soils at different sample points.

Sampling Points	Slope Position	Soil Depth (cm)	Soil Moisture Regime	Soil Structure	Plant Root	Animal Activity	Impact of Human Activities	Intrusions
P1	Top of hill	0–15	Slightly wet	Blocky structure	A few coarse roots, many medium roots	Few earthworms	Mechanical ploughing and planting	Rubbles
P2	shoulder of hill	0–25	wet	Aggregate structure	Few coarse roots, fine roots.	Ant colony	Artificial farming	Stones and rubbles
P3	Waist of hill	0–72	wet	Granular structure	Few fine roots	-	Mechanical ploughing and planting	-
P4	Foot of hill	0–90	Slightly wet	Blocky structure	A few medium roots	-	Mechanical ploughing and planting	Rubbles
P5	Valley of hill	>100	wet	Blocky structure	Many fine roots	Few earthworms, centipedes	Slight disturbance of vegetation	Stones and rubbles

## References

[1] Chen L.M., Zhang G.L., Effland W.R. (2011). Soil characteristic response times and pedogenic thresholds during the 1000-year evolution of a paddy soil chronosequence. Soil Sci. Soc. Am. J..

[2] Goswami S.B., Mondal R., Mandi S.K. (2019). Crop residue management options in rice-rice system: A review. Arch. Agron. Soil Sci..

[3] Kögel-Knabner I., Amelung W., Cao Z.H., Fiedler S., Frenzel P., Jahn R., Kalbitz K., Kölbl A., Kölbl M. (2010). Biogeochemistry of paddy soils. Geoderma.

[4] Mueller-Niggemann C., Utami S.R., Marxen A., Mangelsdorf K., Bauersachs T., Schwark L. (2016). Distribution of tetraether lipids in agricultural soils—Differentiation between paddy and upland management. Biogeosciences.

[5] Timsina J., Connor D.J. (2001). Productivity and management of rice-wheat cropping systems: Issues and challenges. Field Crops Res..

[6] Xue B., Huang L., Huang Y.N., Zhou F.L., Li F., Kubar K.A., Li X.K., Lu J.W., Zhu J. (2019). Roles of soil organic carbon and iron oxides on aggregate formation and stability in two paddy soils. Soil Tillage Res..

[7] FAO (Food and Agricultural Organization of the United Nations) (2009). OECD–FAO Agricultural Outlook.

[8] Wang W., Lai D.Y.F., Wang C., Pan T., Zeng C. (2015). Effects of rice straw incorporation on active soil organic carbon pools in a subtropical paddy field. Soil Tillage Res..

[9] Cui J., Li Z.X., Liu Z.T., Ge B.M., Fang C.M., Zhou C.L., Tang B.P. (2014). Physical and chemical stabilization of soil organic carbon along a 500-year cultivated soil chronosequence originating from estuarine wetlands: Temporal patterns and land use effects. Agric. Ecosyst. Environ..

[10] Bräuer T., Grootes P.M., Nadeau M.J. (2013). Origin of subsoil carbon in a Chinese paddy soil chronosequence. Radiocarbon.

[11] Cao Z.H. (2015). Origin and Evolution of Irrigated Rice Fields and Related Ancient and Present Paddy Soil’s Auality in China.

[12] Rachman A., Anderson S.H., Gantzer C.J., Thompson A.L. (2003). Influence of long-term cropping systems on soil physical properties related to soil erodibility. Soil Sci. Soc. Am. J..

[13] Wei Y.J., Wu X.L., Xia J.W., Miller G.A., Cai C.F., Guo Z.L., Hassanikhah A. (2019). The effect of water content on the shear strength characteristics of granitic soils in South China. Soil Tillage Res..

[14] Wuddivira M.N., Stone R.J., Ekwue E.I. (2013). Influence of cohesive and disruptive forces on strength and erodibility of tropical soils. Soil Tillage Res..

[15] Zhang B., Dang J. (2006). Soil Mechanics and Foundation China.

[16] Horn R. (2003). Stress-strain effects in structured unsaturated soils on coupled mechanical and hydraulic processes. Geoderma.

[17] Rahardjo H., Ong B.H., Leong E.C. (2004). Shear strength of a compacted residual soil from consolidated drained and constant water content triaxial tests. Can. Geotech. J..

[18] Hoyos L.R., Velosa C.L., Puppala A.J. (2014). Residual shear strength of unsaturated soils via suction-controlled ring shear testing. Eng. Geol..

[19] Rahardjo H., Satyanaga A., Leong E.C., Ng Y.S., Pang H.T.C. (2012). Variability of residual soil properties. Eng. Geol..

[20] Fasinmirin J.T., Olorunfemi I.E., Olakuleyin F. (2018). Strength and hydraulics characteristics variations within a tropical Alfisol in Southwestern Nigeria under different land use management. Soil Tillage Res..

[21] Li Q.K. (1992). Paddy Soils of China.

[22] Han Z., Wang X.Y., Song D.D., Li X.X., Huang P., Ma M.H. (2019). Response of soil erosion and sediment sorting to the transport mechanism on a steep rocky slope. Earth Surf. Process. Landf..

[23] Wei C.F., Ni J.P., Gao M., Xie D.T., Hasegawa S. (2006). Anthropic pedogenesis of purple rock fragments in Sichuan Basin, China. Catena.

[24] Dane J., Topp G. (2002). Methods of Soil Analysis Part 4 Physical Methods.

[25] Zhong S.Q., Zhong M., Wei C.F., Zhang W.H., Hu F.N. (2016). Shear strength features of soils developed from purple clay rock and containing less than two-millimeter rock fragments. J. Mt. Sci..

[26] Guo N., Zhao J. (2013). The signature of shear-induced anisotropy in granular media. Comput. Geotech..

[27] Alonso E.E., Pereira J.M., Vaunat J., Olivella S. (2010). A microstructurally based effective stress for unsaturated soils. Geotechnique.

[28] Sadek M.A., Chen Y., Liu J. (2011). Simulating shear behavior of a sandy soil under different soil conditions. J. Terramech..

[29] Wei J., Shi B.L., Li J.L., Li S.S., He X.B. (2018). Shear strength of purple soil bunds under different soil water contents and dry densities: A case study in the Three Gorges Reservoir Area, China. Catena.

[30] Vannoppen W., Vanmaercke M., De Baets S., Poesen J. (2015). A review of the mechanical effects of plant roots on concentrated flow erosion rates. Earth Sci. Rev..

[31] Al-Shayea N.A. (2001). The combined effect of clay and moisture content on the behavior of remolded unsaturated soils. Eng. Geol..

[32] Hu F.N., Liu J.F., Xu C.Y., Wang Z.L., Liu G., Li H., Zhao S.W. (2018). Soil internal forces initiate aggregate breakdown and splash erosion. Geoderma.

[33] Hu F.N., Xu C.Y., Li H., Li S., Yu Z.H., Li Y., He X.H. (2015). Particles interaction forces and their effects on soil aggregates breakdown. Soil Tillage Res..

[34] Hu F.N., Wei C.F., Xu C.Y., Wei N.Q., Zhong M., Zhong S.Q. (2013). Water sensitivity of shear strength of purple paddy soils. Trans. CSAE.

[35] Mitchell J.K., Soga K. (2005). Fundamentals of Soil Behavior.

[36] Kwan A.K.H., Fung W.W.S. (2012). Roles of water film thickness and SP dosage in rheology and cohesiveness of mortar. Cem. Concr. Compos..

